# Postproline Cleaving Enzymes also Show Specificity to Reduced Cysteine

**DOI:** 10.1021/acs.analchem.4c04277

**Published:** 2024-11-19

**Authors:** Zuzana Kalaninová, Jasmína Mária Portašiková, Barbora Jirečková, Marek Polák, Jana Nováková, Daniel Kavan, Petr Novák, Petr Man

**Affiliations:** † Department of Biochemistry, Faculty of Science, Charles University, Hlavova 6, Prague 2 12843, Czechia; ‡ Institute of Microbiology of the Czech Academy of Sciences, BioCeV, Videnska 1083, Prague 4 14220, Czechia; § AffiPro s.r.o., Nad Safinou II 366, Vestec 252 00, Czechia

## Abstract

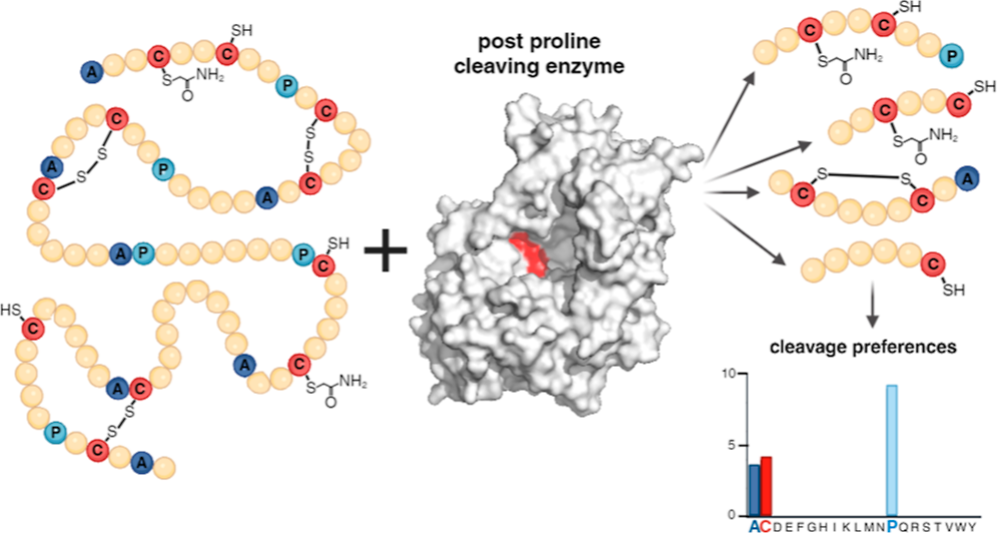

In proteomics, postproline cleaving enzymes (PPCEs), such as *Aspergillus niger* prolyl endopeptidase (*An*PEP) and neprosin, complement proteolytic tools because proline is a stop site for many proteases. But while aiming at using *An*PEP in online proteolysis, we found that this enzyme also displayed specificity to reduced cysteine. By LC–MS/MS, we systematically analyzed *An*PEP sources and conditions that could affect this cleavage preference. Postcysteine cleavage was blocked by cysteine modifications, including disulfide bond formation, oxidation, and alkylation. The last modification explains why this activity has remained undetected so far. In the same experimental paradigm, neprosin mimicked this cleavage specificity. Based on these findings, PPCEs cleavage preferences should be redefined from post-Pro/Ala to post-Pro/Ala/Cys. Moreover, this evidence demands reconsidering PPCEs applications, whether cleaving Cys-rich proteins or assessing Cys status in proteins, and calls for revisiting the proposed enzymatic mechanism of these proteases.

## Introduction

Proteolysis is a key step in proteomics and MS-based structural analysis of proteins by H/D exchange (HDX), chemical cross-linking, and radical labeling.^1−3^ By digesting proteins into shorter peptides, proteolysis facilitates MS analysis and data processing, overcoming limitations inherent to intact protein analysis. However, proteolysis is incorporated into methods exploring a large search space during data analysis, whether in proteomics, which examines whole cells with thousands of proteins, or protein structural MS analysis, which introduces complexity through heterogeneous reaction products. To avoid introducing additional levels of complexity when leveraging proteolysis, these workflows rely on specific enzymes with known digestion patterns such as trypsin.

Trypsin specifically and efficiently cleaves proteins after Arg and Lys, with minimal missed sites, yielding predictable peptides. Because the C-terminal basic residues of these peptides favor ionization, the resulting rich fragment ion series streamline database searches and peptide identification.^4−6^ Trypsin is also quite affordable, so trypsinization has become the gold standard of proteome digestions. Yet, despite its reliability and numerous advantages,^5^ unwavering research interest in alternative enzymes for proteomics^1^ has provided access to proteins with an unfavorable Lys and Arg distribution^1,5,7,8^ and with post-translational modifications.^6,9,10^ Proteases like Asp-N, Glu-C, Lys-C, or Arg-C offer the advantage of a variable distribution of cleavage sites, and their utilization may enhance proteome coverage.^1,5,11^ Proteases with N-terminal specificity, such as Tryp-N and LysargiNase, generate N-terminally charged peptides, predominantly forming b-ion series upon MS/MS fragmentation and, hence, simplifying spectral pattern recognition.^10,12^ Among these promising enzymes, two postproline cleaving enzymes (PPCEs) stand out for their highly selective cleavage after proline and alanine,^13^ namely, neprosin^8,14^ and *Aspergillus niger* prolyl endopeptidase (*An*PEP).^15,16^

Neprosin is a prolyl endopeptidase discovered in the pitcher fluid of tropical carnivorous plants.^17−19^ Produced as a zymogen, neprosin consists of two domains—the neprosin activation peptide and the catalytic domain.^19^ The active site of the substrate cleft contains two highly conserved Glu residues, and as with other glutamic peptidases,^19,20^ neprosin is activated at low pH via autoproteolysis, preferentially cleaving proteins C-terminally to Pro and Ala at low concentrations.^8,19^ This PPCE cleaves toxic peptides generated from gluten under gastric conditions, thus meeting the criteria for a therapeutic glutenase.^14^ Furthermore, when tested for whole-proteome analysis and histone mapping,^8^ neproin shows no protein substrate size bias, mirroring *An*PEP.

*An*PEP is an acidic prolyl endopeptidase produced by the filamentous fungus *A. niger*, and its application in proteomics was first described by Sebela et al. in 2009.^13,21^ It belongs to the serine peptidase family S28 of ProXaa carboxypeptidases, primarily cleaving proteins on the C-terminal side of Pro and Ala residues.^20,22^ For its unique selectivity, *An*PEP has benefitted the brewing industry^23^ and spurred therapeutics^17,24^ for celiac disease by targeting Pro-rich substrates. Although the structure–function relationship of its different proteoforms remains mostly unknown,^25^*An*PEP can digest large substrates, unlike other prolyl endopeptidases with a substrate size limited to 30 residues.^20^ In proteomics applications,^7^*An*PEP processes several analytical targets too challenging for trypsin, including histones (for PTM mapping) or ancient mammoth bone (for protein identification). Its ability to withstand and be active even at very low pH (1.5–2.5)^7,15^ is crucial for analyses like disulfide bond mapping in monoclonal antibodies or HDX.

Considering the above, we aimed at preparing and testing an *An*PEP-based column for online digestion, thereby inadvertently unveiling the high selectivity of this enzyme for cleavage after reduced cysteine. Since *An*PEP specificity can be fine-tuned by tightly controlling digestion conditions,^7^ we tested different protease sources at various conditions. Regardless of the source, *An*PEP displayed the same specificity, and its selectivity did not derive from hidden contaminating proteases. *An*PEP post-Cys selectivity was blocked by modifications such as disulfide bonding, oxidation, and alkylation. Moreover, the same selectivity and sensitivity to Cys modifications was found in neprosin as well. In combination, our findings demonstrate that PPCEs also specifically cleave proteins after reduced cysteine.

## Experimental Section

### Protease and Sample Preparation

Research-grade ProAlanase was purchased from Promega. *A. niger* prolyl endoprotease (*An*PEP) was isolated from Clarity Ferm (White Laboratories). For buffer exchange, a P-10 desalting column packed with Sephadex G-25 resin (GE Healthcare) was washed and equilibrated with water (4 × 4 mL) and sodium citrate buffer (50 mM, pH 5.0; 5 × 4 mL). Subsequently, 2.5 mL of Clarity Ferm was applied and allowed to enter the bed completely. *An*PEP was eluted with 3.5 mL of sodium citrate buffer.

Protein concentration was determined using a Pierce BCA protein assay kit (Thermo Fisher Scientific). Isolated *An*PEP was immobilized on an aldehyde-functionalized POROS 20 AL resin, as described previously.^26−28^ Recombinant neprosin was expressed according to a literature protocol.^8^ Whether for offline or online digestion, the protein mixture in Tris-HCl buffer (100 mM, pH 8.8) consisted of five proteins, namely, bovine serum albumin (BSA), bovine carbonic anhydrase II (bCA2), horse cytochrome C, and horse myoglobin (Mb), which were purchased from Merck Life Sciences, and 14-3-3gamma, which was recombinantly expressed and purified.^29^ The concentration of all proteins was 10 μM, as confirmed using the Pierce BCA protein assay kit.

HEK293 cells were resuspended in a lysis buffer consisting of 100 mM triethylammonium bicarbonate, pH 8.5 and 2% sodium deoxycholate. The cells were denaturated in a thermomixer (2000 rpm) for 5 min at 95 °C prior to sonication. Once the samples were cooled down, protein concentration was determined using the Pierce BCA protein assay kit (Thermo Fisher Scientific). Sodium deoxycholate detergent and other salts were removed by acetone precipitation. Precooled (−20 °C) acetone was added to cell lysates to reach 70% final concentration. After 2 h at −20 °C, the samples were centrifuged at 16,100 ×*g* for 15 min. The protein pellet was resuspended in 20 mM Tris-HCl buffer (pH 8.5), and protein concentration was again determined using the Pierce BCA protein assay kit (Thermo Fisher Scientific).

The protein mixture and cell lysate samples were reduced with 10 mM tris(2-carboxyethyl)-phosphine (TCEP) at 65 °C for 10 min. The Cys alkylated samples were prepared by incubating the reduced samples with 20 mM iodoacetamide (IAA) for 1 h at room temperature in the dark. Human insulin and oxidized bovine beta-insulin chain were purchased from Merck Life Sciences. Serum from healthy donors of the Department of Transfusion Medicine and Blood Bank at Thomayer University Hospital in Prague was diluted (10 ×) with Tris-HCl buffer (100 mM, pH 8.8) and reduced with TCEP (10 mM) for 10 min at 65 °C.

### Insulin Digestion and MALDI-FT-ICR MS Analysis

Insulin was dissolved in 5% acetic acid, and its disulfide bonds were reduced using 50 mM TCEP for 4 h at 21 °C. The oxidized beta chain was dissolved directly in 50 mM Tris-Cl pH 8.0. Nonreduced and reduced insulin and oxidized beta chain were transferred to 30 mM HCl pH 1.5, followed by *An*PEP digestion at a 1:50 enzyme/protein ratio for 2 h at 37 °C. Samples were analyzed on matrix-assisted laser desorption/ionization Fourier transform ion cyclotron resonance mass spectrometry (MALDI-FT-ICR MS; 15T solariXR, Bruker Daltonics) using alpha-cyano-4-hydroxycinnamic acid as the MALDI matrix.

### On-Column Protein Digestion and LC–MS/MS Analysis

Online digestion and analysis were performed in a fully automated mode. The samples were handled by a PAL DHR robot (CTC Analytics AG) controlled by the Chronos software (Axel Semrau). A 5 μL aliquot of reduced protein mixture (50 pmol per injection) was diluted with 95 μL of 30 mM HCl and manually injected into the LC system. To analyze serum samples, 10 μL of reduced serum was diluted with 40 μL of 50 mM HCl and mixed with 50 μL of 30 mM HCl or 4 M urea in 30 mM HCl. The LC system consisted of a custom-made column with immobilized *An*PEP (69 μL bed volume), a trap column (SecurityGuard ULTRA Cartridge UHPLC Fully Porous Polar C18, 2.1 mm; Phenomenex), and an analytical column (Luna Omega Polar C18, 1.6 μm, 100 Å, 1.0 × 100 mm; Phenomenex). Sample digestion and peptide desalting were performed in 30 mM HCl delivered by the 1260 Infinity II Quaternary pump (Agilent Technologies) at 100 (2 or 21 °C) or 200 (2 °C) μL/min. The peptides were separated for 6 min in a water/acetonitrile gradient (5%–45% (v/v); solvent A: 0.1% formic acid (FA) in water, solvent B: 0.1% FA, 2% water in acetonitrile), followed by a step to 99% B delivered by the 1290 Infinity II LC system (Agilent Technologies) at a flow rate of 40 μL/min. Serum samples were analyzed for 27 min in a water/acetonitrile gradient (5%–35% (v/v); solvent A: 0.1% FA in water, solvent B: 0.1% FA, 2% water in acetonitrile), followed by a step to 99% delivered by the 1290 Infinity II LC system. The timsTOF Pro mass spectrometer, with active parallel accumulation-serial fragmentation (PASEF) and 100 ms tims ramp time, was operated in a data-dependent MS/MS mode.

### In-Solution Protein Digestion and Analysis

To test protease activity in solution, 5 μL aliquots of reduced protein mixture (containing 15 μg of protein) were diluted with 95 μL of HCl (30 mM, pH 1.5). Protease (1 μL), either research-grade ProAlanase or purified *An*PEP, was added at a 1:50, 1:100, and 1:200 ratio (w/w). The reactions were performed in triplicate for 1, 2, and 18 h digestion. Additionally, reaction mixtures at 1:5, 1:10, and 1:500 (w/w) enzyme/protein ratios were incubated for 2 h only. An alkylated protein mixture was used to investigate cleavage preferences for cysteine status by digestion for 2 h at a 1:50 (w/w) ratio in triplicate. All digestions were stopped by increasing the pH to 7.0 with 1 M Tris solution. Samples were subsequently analyzed by LC–MS/MS. Peptides were first desalted by 0.4% FA in water driven by an LC-20AD pump (Shimadzu) at 20 μL/min using a Luna Omega (5 μm Polar C18 100 Å, Micro Trap 20 × 0.30 mm) trap column (Phenomenex) and then eluted and separated for 15 min in a water/acetonitrile gradient (5%–35% (v/v); solvent A: 0.1% FA in water, solvent B: 0.1% FA, 2% water in acetonitrile), followed by a step to 99% solvent B driven by the Agilent 1200 HPLC system at a flow rate of 4 μL/min using a Luna Omega (3 μm Polar C18 100 Å, LC Column 150 × 0.30 mm) analytical column (Phenomenex) heated to 50 °C. The LC system was directly connected to an ESI source of the timsTOF Pro mass spectrometer with enabled PASEF (Bruker Daltonics), operating in a data-dependent MS/MS mode with a 100 ms tims ramp time. To test protease activity on a more complex sample, 10 μL (10 μg of protein content) aliquot of reduced HEK293 cell lysate was diluted with 90 μL of 30 mM HCl (pH 1.5). Either research-grade ProAlanase or purified *An*PEP (1 μL) was added at a 1:50 (w/w) ratio. The reactions were stopped after 2 h at 37 °C by increasing the pH to 7.0 with a 1 M Tris solution. The peptides were desalted, eluted, and separated for 44 min in a water/acetonitrile gradient (4%–35% (v/v); solvent A: 0.1% FA in water, solvent B: 0.1% FA, 20% water in acetonitrile), followed by a step to 99% solvent B driven by the UHPLC system (VanquishTM Neo, Thermo Scientific) at a 1.5 μL/min flow rate. The system consisted of a PepMap Neo (5 μm C18, 50 × 0.30 mm, Thermo Scientific) trap cartridge and a PepSep (1.5 μm C18, 150 × 0.15 mm, Bruker Daltonics) analytical column heated to 50 °C. The outlet was directly coupled to an ESI source of a timsTOF SCP mass spectrometer (Bruker Daltonics), operating in a data-dependent mode employing PASEF. To test neprosin (another post-Pro/Ala protease) for its preference for Cys, recombinant protease was added to 5 μL of protein mixture diluted with glycine (50 mM, pH 2.5) at 1:50, 1:100, and 1:200 (w/w) ratios, and the reactions were performed for 2 h at 37 °C in triplicate. The pH was raised to 7.0 with 1 M Tris solution. The samples were analyzed by LC–MS/MS on a timsTOF SCP mass spectrometer (Bruker Daltonics) identically as described above, with the exception for gradient length which was 15 min in this case.

### *An*PEP Profiling by LC–MS/MS

In its native form or after Endo H deglycosylation (50 mM Bis-Tris-Cl pH 6.0, 37 °C, 18 h), Clarity Ferm *An*PEP was transferred into 50 mM ammonium bicarbonate and subjected to in-solution digestion by either trypsin or Glu-C. Cys residues were blocked by reduction with 10 mM TCEP and by alkylation with 20 mM IAA. The peptides were injected onto a PepMap Neo (5 μm C18, 50 × 0.30 mm, Thermo Scientific) trap cartridge, where they were desalted with 0.1% FA in water and separated on a PepSep (1.5 μm C18, 150 × 0.15 mm) analytical column for 15 min by a water/acetonitrile gradient (4%–35% (v/v); solvent A: 0.1% FA in water, solvent B: 0.1% FA, 20% water in acetonitrile). The column was kept at 50 °C. Solvents were delivered by the UHPLC system (VanquishTM Neo, Thermo Scientific) at a 1.5 μL/min flow rate. The outlet of the LC was directly coupled to an ESI source of the timsTOF SCP mass spectrometer (Bruker Daltonics), operating in a data-dependent mode employing PASEF.

### Data Processing

LC–MS/MS data were peak-picked in DataAnalysis 5.3 software (Bruker Daltonics), exported to *.mgf files, and searched using MASCOT (version 2.7, Matrix Science) against a custom-built database combining the sequences of the proteins in the mixture with a common cRAP.fasta (https://www.thegpm.org/crap/) and the sequences of the proteases. Serum digestion data were searched against the UniProt/Swiss-Prot database (release 2023_01). *An*PEP profiling data were searched against a subset of the trEMBL database containing all proteins from *Aspergillus* sp. and a specific sequence of *An*PEP from GenBank (GAQ41715.1). In all cases, the MASCOT search parameters were set to 10 ppm precursor tolerance, 0.05 Da fragment ion tolerance and decoy search enabled, with FDR <1%, IonScore >25, and peptide length >6. Variable modifications: Cys—Heme or Dehydro and protein N-term-acetylation were used for protein mixture. Cys carbamidomethylation was added for alkylated samples. *An*PEP profiling data included Cys carbamidomethylation as a fixed modification, while protein N-term acetylation and HexNAc at Asn were entered as variable modifications. Full MASCOT search results were exported as *.csv files. HEK293 cell lysate samples were searched using the PEAKS Studio software v12 against a human UniProt database and the following search parameters: no enzyme, 10 ppm mass tolerance for precursor, and 0.05 Da for fragment ions. The modifications were set as follows: variable HexNAc at Asn and fixed Cys carbamidomethylation for alkylated samples. Decoy search was enabled, and the result was filtered at FDR <1%. Digest parameters—average redundancy, redundancy distribution, sequence coverage, number of unique peptides, average peptide length, and cleavage preferences—were evaluated using a new in-house developed Java-based software tool, DigDig. For cleavage preferences, nonredundant cleavage sites were extracted from the MASCOT or PEAKS search results. Data were normalized for the occurrence of individual amino acids in the identified sequences, according to Keil.^30^

## Results and Discussion

Following the recent description of *An*PEP/ProAlanase as a specific enzyme,^7^ we aimed to generate *An*PEP-based proteolysis columns. To this end, we systematically compared *An*PEP from two sources—Clarity Ferm (solution-based) and Tolerase G capsules—that is, industrial and food-supplement-grade *An*PEP, respectively (Figure S1). For its cleaner profile and easier handling, we selected the Clarity Ferm-based material for standard immobilization on a POROS 20 AL resin.

Immobilized protease was packed into a column and used in a typical HDX LC–MS/MS setup, replacing the digestion/desalting solution with 30 mM hydrochloric acid to lower the pH to 1.5, which is crucial for enzyme specificity.^7^ A prereduced, equimolar mixture of five purified, intact proteins differing in proline content and size was selected as the test sample. Proteolysis was performed online, at 21 °C and 100 μL/min flow rate, which is typically used for a proteolytic column of this size.^31^ However, specificity was lower than expected, so we restricted the digestion conditions by modifying the flow rate and temperature. More specifically, we performed digestion at a low temperature (2 °C) to slow down protease activity and then reduced the digestion time by increasing the flow rate to 200 μL/min.

**Figure 1:**
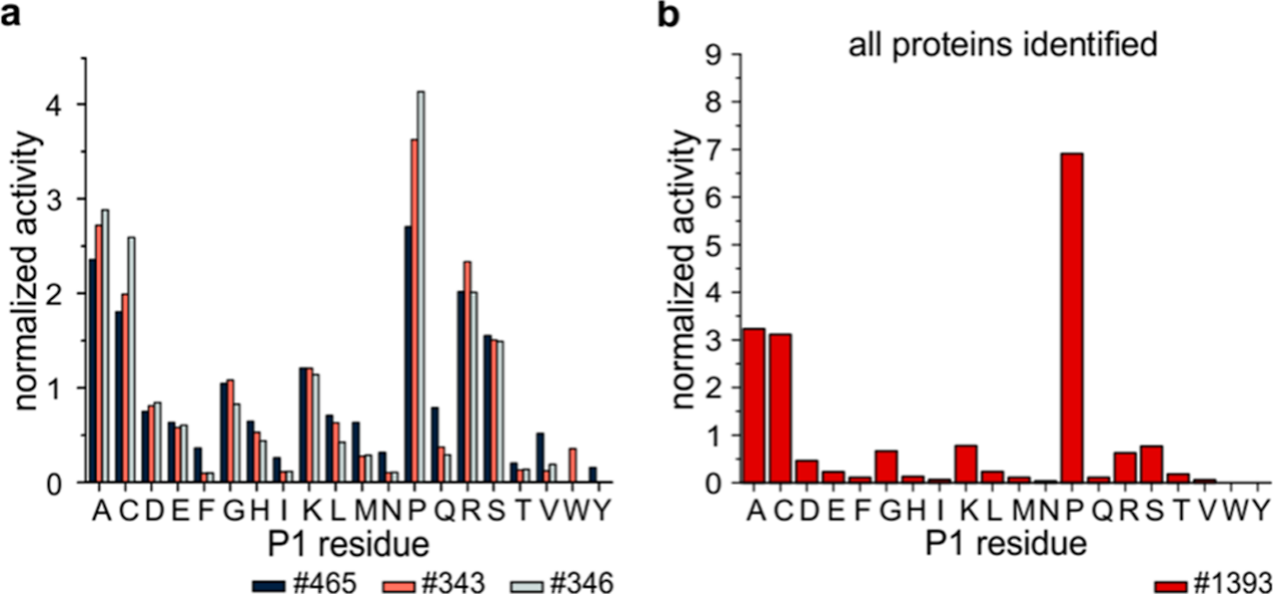
Analysis of cleavage preferences of immobilized *An*PEP. *An*PEP selectivity (P1 position—cleavage after each residue) based on fully reproducible peptides resulting from online digestion of (a) protein mixture at different flow rates and/or temperatures (black bars—100 μL/min, 21 °C, orange—100 μL/min, 2 °C, and gray—200 μL/min, 2 °C) and (b) prereduced human serum (digestion at 100 μL/min, 2 °C). The estimated online digestion times are 41 s (at 100 μL/min) and 21 s (at 200 μL/min). A number of peptides used in the individual conditions are provided below the graphs; the underlying data are available as Table S1.

Digestion reached at least 80% sequence coverage but a rather poor selectivity to Pro and Ala (Figure S2a). Only when full reproducibility (6 of 6) was considered did the preferences center more around Pro and Ala (Figure 1a). However, we noticed a remarkably high preference for cleavage after Cys, Arg, Ser, and, quite frequently, Gly, Lys, and Asp. Even under more stringent digestion conditions (faster flow + lower temperature), the cleavage preferences were still far from the pattern previously shown in in-solution digestion.^7^

To test the cleavage preferences in a more complex matrix and to probe the high selectivity for cleavage after Cys, we used prereduced human serum from a healthy donor. Here, only the most abundant protein, serum albumin, reached full coverage, which was otherwise lower than 50% for other proteins. The cleavage preferences for fully reproducible peptides were centered much more around post-Pro and Ala (Figure 1b) than those for a simple protein mixture. We also extracted preferences for the top, fully covered serum albumin (Figure S2b) and for all remaining proteins (Figure S2c). The cleavage preferences for albumin alone were much less selective, resembling those of the test mixture. Nevertheless, HSA subtraction from the serum protein pool further increased the specificity. This result clearly demonstrates bias in assessing preferences using complex protein mixtures, which may lead to overestimating protease/*An*PEP specificity. In other words, data from complex mixtures composed of only partial protein coverages cannot be easily extrapolated to single proteins, at least not for the Clarity Ferm-based *An*PEP column.

**Figure 2:**
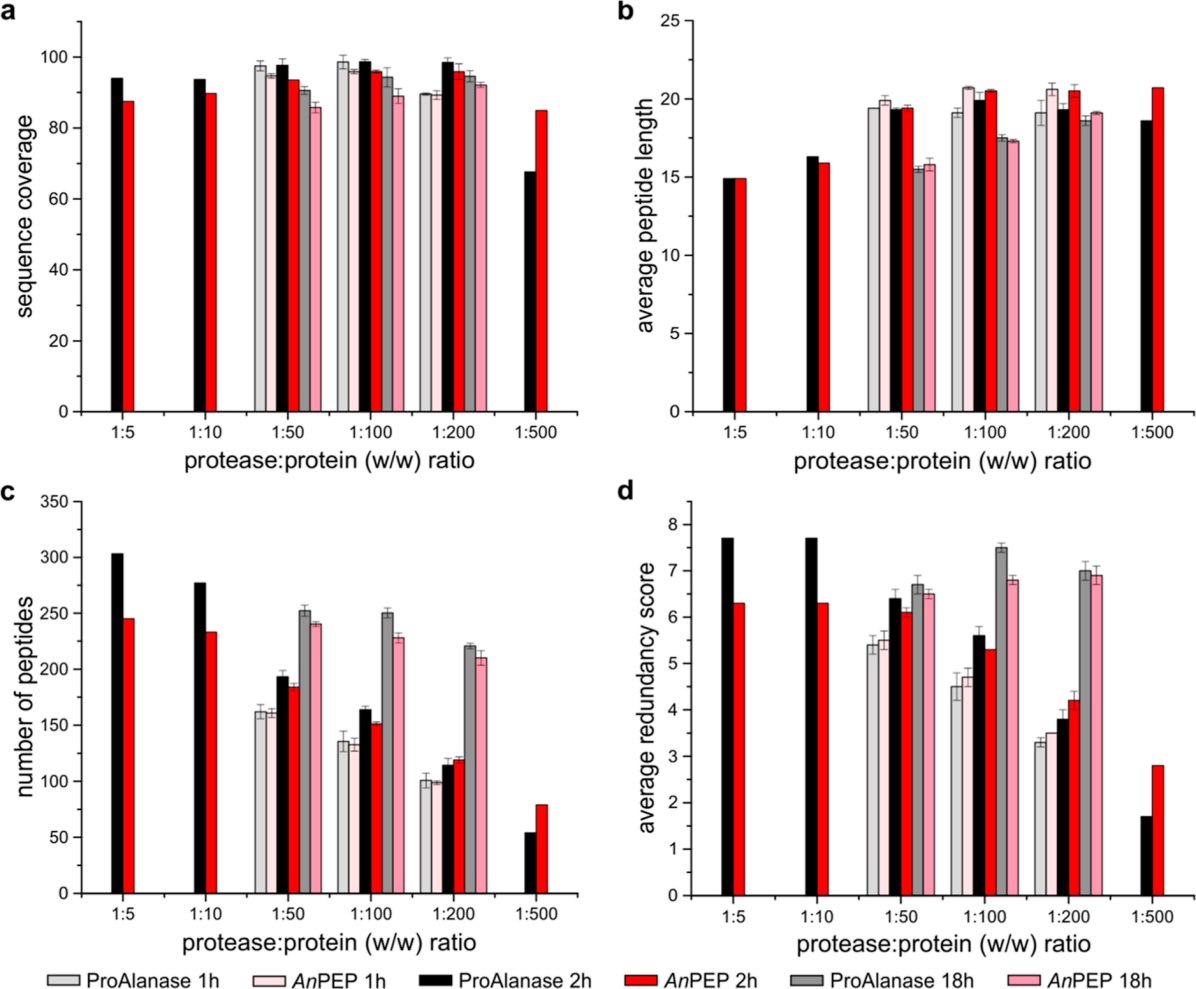
Comparison of key digestion parameters for ProAlanase and Clarity Ferm *An*PEP. Using the nonredundant peptides from BSA (component of the test protein mixture), four parameters were extracted, namely, (a) sequence coverage, (b) average peptide length, (c) number of peptides, and (d) average redundancy, and plotted at enzyme/protein ratios ranging from 1:5 to 1:500. ProAlanase and Clarity Ferm *An*PEP are highlighted in black/gray and red/pink hues, respectively, representing 1, 2, and 18 h digestion times.

The high selectivity for cysteine was particularly intriguing. To determine whether the lower selectivity and strong post-Cys preferences of immobilized *An*PEP were specific properties of the Clarity Ferm-based material or caused by immobilization, we compared *An*PEP digestion in solution with MS-grade ProAlanase digestion of a prereduced protein mixture at three enzyme/protein (w/w) ratios (1:50, 1:100, and 1:200) and digestion times (1, 2, and 18 h). As previously suggested,^7^ the optimal digestion time of 2 h was then used to probe additional conditions—1:5, 1:10, and 1:500 enzyme/protein ratios. To parametrize the digestions, we used all the nonredundant peptides that were identified from the largest protein in the mixture—BSA—because this protein provided enough peptides under all conditions tested in this study and, thus, can be considered a statistically relevant representative of the digestion. The data showed a good agreement between the MS-grade enzyme and the Clarity Ferm *An*PEP, as shown in plots comparing different digestion parameters (Figure 2).

Under virtually all conditions tested in this study, the sequence coverage was high, mostly above 80%, and similar. Only the lowest enzyme/protein ratio of ProAlanase produced lower coverage. The two enzymes also showed agreement in terms of average peptide length, with short peptides appearing at the highest enzyme/protein ratio and/or longer digestion times. The number of peptides and average redundancy were also similar at 1:50, 1:100, and 1:200 ratios, with slight differences only at higher ratios, where ProAlanase generated more peptides, which led to higher redundancy. Otherwise, the enzymes were highly comparable and followed the same logical trends.

**Figure 3:**
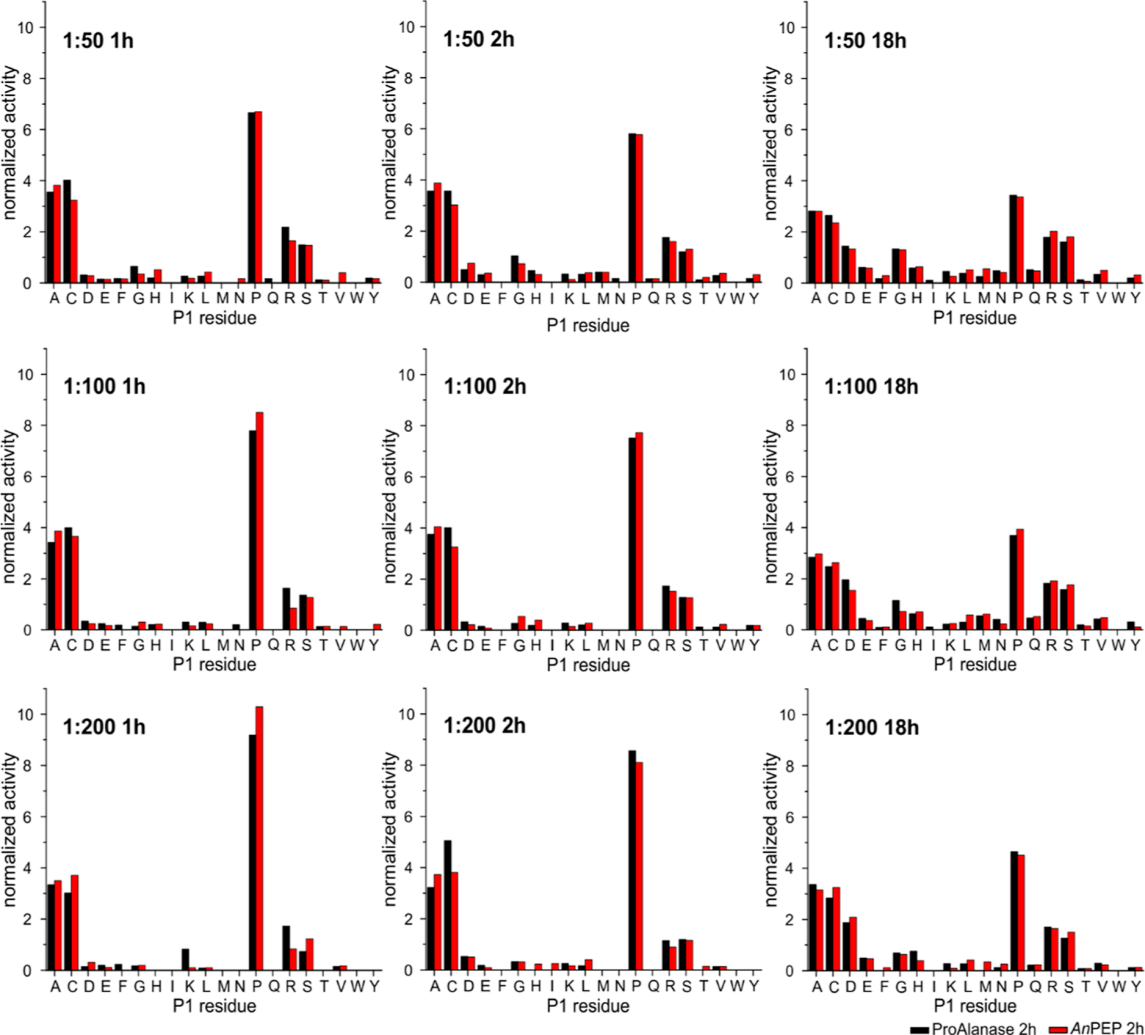
Comparison of cleavage preferences of Clarity Ferm *An*PEP and research-grade ProAlanase in solution. An equimolar mixture of five prereduced, purified intact proteins was digested by ProAlanase and Clarity Ferm *An*PEP at various digestion parameters—1, 2, or 18 h time and 1:5—1:500 enzyme/protein ratios. Cleavage preferences at the P1 site (digestion after the residue) are shown for ProAlanase (black) and Clarity Ferm *An*PEP (red) as a function of digestion parameters, indicated in the top left corner of each graph, highlighting a clear trend in specificity, which increases with the decrease in the enzyme/protein ratio and decreases with the digestion times. Preferences at the P1′ site are shown in Figure S3. The data are based on fully reproducible peptides (3 of 3).

**Figure 4:**
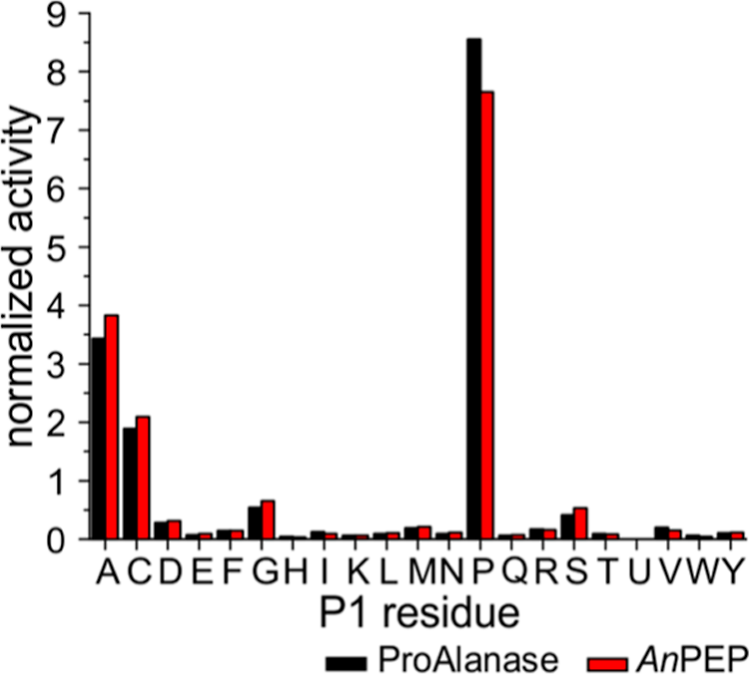
Comparison of cleavage preferences of Clarity Ferm *An*PEP and research-grade ProAlanase in a whole-cell lysate. The whole-cell lysate (HEK293 cell line) was prereduced by TCEP and subjected to in-solution digestion for 2 h, at pH 1.5 and 37 °C. Cleavage preferences are based on fully reproducible peptides (3 of 3).

When comparing cleavage preferences as a function of digestion time and protease ratio, we found no major differences between ProAlanase and Clarity Ferm *An*PEP. For both enzymes, the specificity was largely post-Pro/Ala, with significant post-Cys cleavage (Figures 3, S3, and S4). Both enzymes also followed similar trends: specificity decreased at a longer incubation time, especially overnight (18 h), and with the increase in enzyme/protein ratios, corroborating previously published data for ProAlanase. These findings were confirmed at the optimal digestion time of 2 h^7^ and low (1:500) and high (1:5 and 1:10) ratios (Figure S4), showing that a high ratio—high protease concentration—decreases digestion specificity and, thus, likely explains our online digestion observations given the high local amount of the enzyme. Therefore, the lower specificity of the immobilized *An*PEP can be attributed to higher protease loading rather than to less specific properties of the Clarity Ferm-based enzyme (see Figure 4).

The enzyme/resin ratio may be lowered to produce a more specific column, but this approach would require further fine-tuning as a less dense protease column may also lead to incomplete digestion. No significant trend or preference was observed in the P1′ position (Figures S3 and S4b), except for the relatively high pre-Cys tendency, most likely due to sequence specificities of serum albumin, the main source of Cys in the protein mixture, with eight Cys pairs in the primary sequence. Since Cys is one of the highly preferred amino acids in position P1, post-Cys cleavage is more frequent than after other sites and, accordingly, so is Cys–Cys digestion, explaining the higher pre-Cys preference.

To address the role of sample complexity and to better compare our data with previous results,^7^ we subjected a prereduced, whole-cell lysate (human cell line HEK293) to in-solution digestion with ProAlanase and Clarity Ferm *An*PEP for 2 h, at pH 1.5 and 37 °C. For both enzymes, the results were highly comparable and confirmed that they cleave primarily after Pro, followed by post-Ala and post-Cys. In addition, using a more complex mixture attenuated nonspecific cleavages, whereas extracting cleavage preferences from proteins with a high sequence coverage (such as Hsp70, actin, alpha-enolase, and HNRNP) highlighted the extent of nonspecific cleavage.

To uncover any potential protease contamination, we performed proteomic profiling of Clarity Ferm *An*PEP. Based on the peptides identified from each protein (Tables S2 and S3), *An*PEP was the main component, corroborating the SDS-PAGE profile (Figure S1). Other minor components included alpha-amylase, GPI-anchored cell wall organization protein Ecm33, Isomerase YbhE, and importantly also serine carboxypeptidase. This serine carboxypeptidase could not be the source of the post-Cys cleavage because the content of this low-level contaminant was much lower than that of *An*PEP, and it can only account for ragged C-terminal ends, not for peptides derived from endopeptidase action such as those in data sets with Cys preceding their N-terminal side. However, serine carboxypeptidase may contribute to the nonspecific pattern at higher enzyme/protein ratios. Accordingly, the lower specificity of the enzymatic columns may also be partly caused by this contaminant, even though most of the nonpost-Pro/Ala/Cys cleavages are likely be attributed to the high local protease concentration.

To assess how pH affects cleavage patterns of *An*PEP, digestions were performed at pH 4.5 for either 2 or 18 h. The main preferences remained directed toward Pro, Ala, and Cys (Figure S5), ruling out any pH-related effects. Nevertheless, increasing the pH decreased the frequency of cleavages after aspartic acid, demonstrating that a substantial portion of these events occur via acid hydrolysis, not enzymatic action. For this reason, digestions at very low pH should be performed either online or for shorter times than normally used for trypsin or other specific proteases working at pH values ranging from neutral to slightly basic.^32,33^

**Figure 5:**
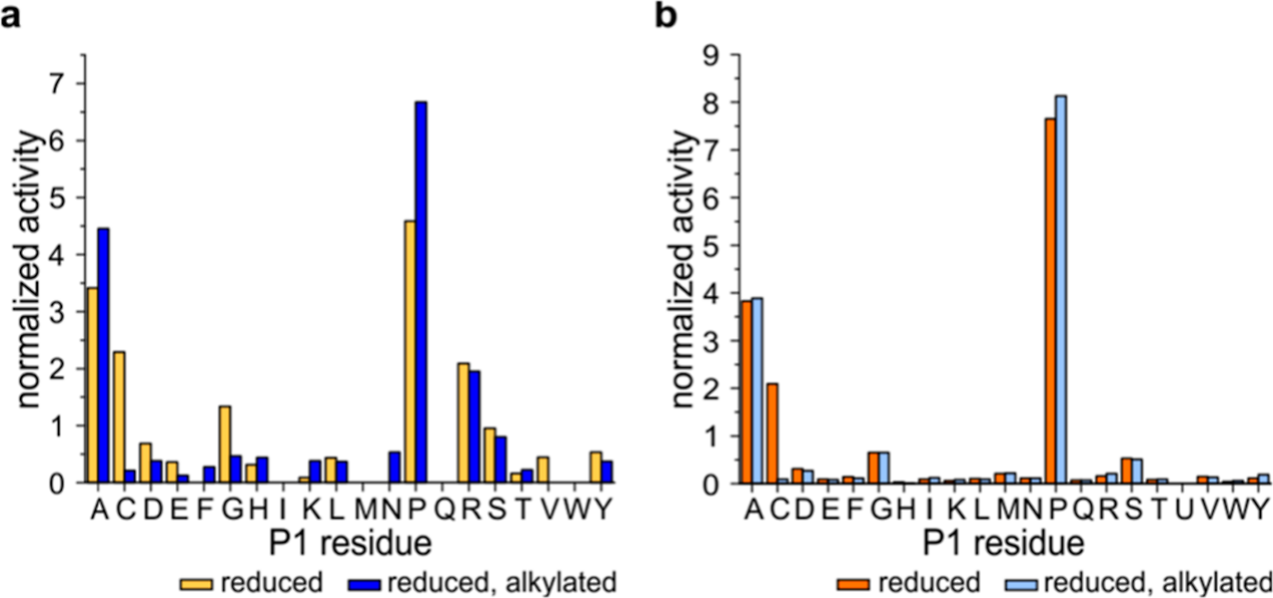
*An*PEP targets cysteine residues only in their reduced form. The effect of Cys alkylation on the digestion pattern was tested on (a) test protein mixtures and (b) HEK293 cell lysates. Prereduced and IAA-alkylated samples were digested with Clarity Ferm *An*PEP at pH 1.5 and 37 °C for 2 h. Both samples show a clear drop in post-Cys cleavage after alkylation, followed by an increase in post-Ala and post-Pro specificity, especially in the mixture of purified intact proteins.

In the next step, we evaluated how Cys status affects the post-Cys cleavage. When comparing the cleavage patterns of reduced and Cys-alkylated protein mixtures or HEK293 cell lysate, we identified a sharp decrease in post-Cys cleavage and a concomitant increase in post-Ala and post-Pro cleavages (Figure 5). These results explain why the post-Cys preference had not been identified before because all previous studies have used protein reduction and alkylation.^7,15,25^

**Figure 6:**
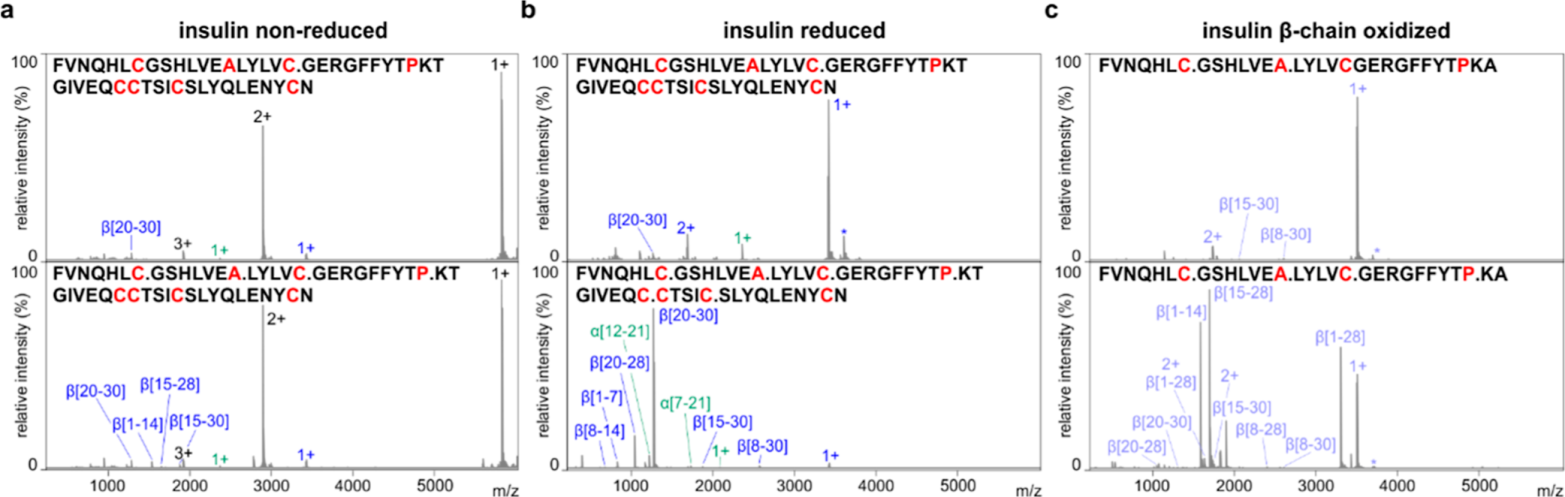
Validation of *An*PEP cleavage of reduced Cys using insulin as a target. *An*PEP digestion of (a) nonreduced and (b) reduced human insulin and (c) oxidized bovine insulin beta chain. Spectra before (top) and after (bottom) *An*PEP digestion for 2 h at 37 °C and a 1:50 enzyme/protein ratio (w/w). Intact insulin is highlighted in black; fragments from the alpha chain, in green; and fragments from the beta chain, in blue.

As a probe for *An*PEP post-Cys activity on Cys either involved in disulfide bonds or oxidized, we selected insulin. Insulin has 6 Cys residues forming three disulfide bonds and only one Ala and one Pro in the beta subunit. In-solution digestion of nonreduced insulin analyzed by MALDI-FT-ICR showed (Figure 6a) poor proteolysis, most likely also due to the compact structure of insulin, with minor fragments arising from digestion after Pro and Ala. Conversely, upon reduction, both chains were cleaved at Cys sites (Figure 6b). Upon digestion of oxidized bovine beta insulin chain (Figure 6c), we observed that oxidation blocks *An*PEP cleavage similarly to alkylated Cys and that the primary cleavage sites are Pro and Ala. Such findings support previous claims that *An*PEP can be used to map disulfide bonds in proteins and further extend the potential use of *An*PEP as a validation tool for probing disulfide bond status or Cys modifications, including oxidation and acylation.^7^

**Figure 7:**
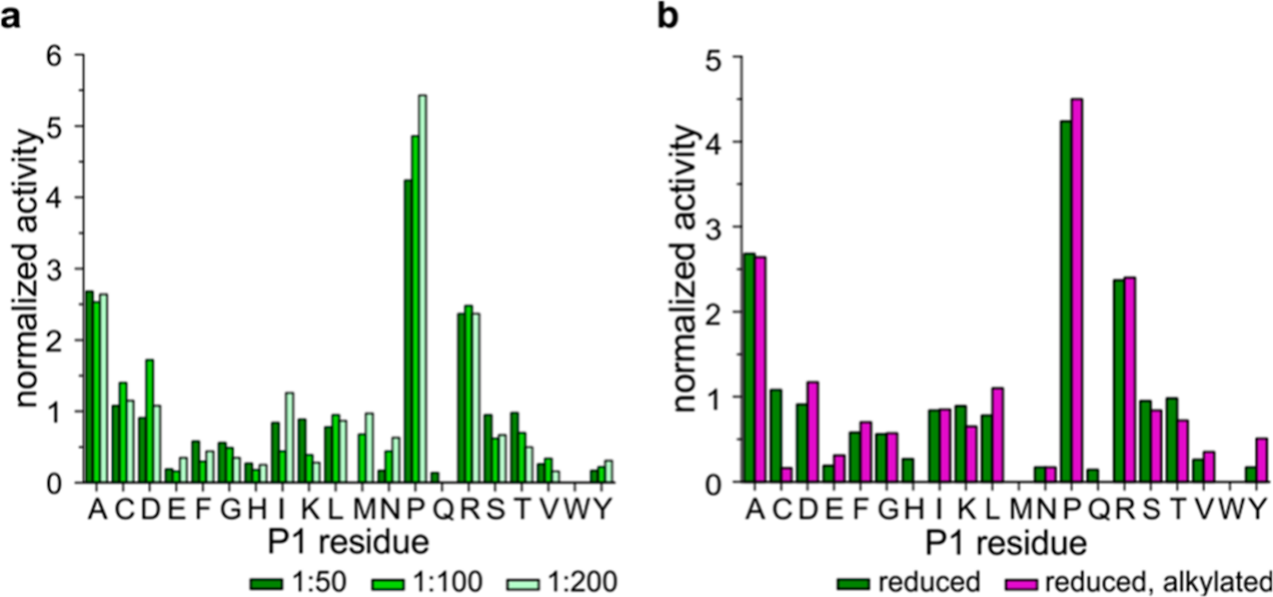
Neprosin cleavage preferences include cysteine and are blocked by alkylation. (a) Cleavage preferences of neprosin followed at various enzyme/protein ratios. (b) Neprosin post-Cys cleavage is also blocked by alkylation, as shown by digestion of reduced (green) and alkylated (purple) protein mixtures.

Assuming that Cys cleavage was overlooked in *An*PEP for the same reason (Cys alkylation) as in other enzymes with similar cleavage specificities, we tested recombinant neprosin, which is a glutamate endopeptidase.^8,14,17,19^ Due to its pH optimum,^8^ we modulated the conditions slightly and performed digestion at pH 2.5 for 2 h at 1:50, 1:100, and 1:200 enzyme/protein ratios. Neprosin applied to a simple protein mixture showed slightly worse specificity than expected (Figure 7a) when post-Arg and Asp appeared at a rather high frequency, alongside with post-Pro, Ala, and Cys. Nevertheless, the main question regarding post-Cys preferences was clearly answered, and the results were confirmed when digesting an alkylated sample (Figure 7b). The clear drop in post-Cys activity corroborated the results from our *An*PEP experiments, demonstrating that alkylated Cys is not cleaved by post-Pro/Ala selective proteases.

These findings not only reveal new *An*PEP and neprosin specificity but also flag a bias in the analysis of enzyme preferences based on proteomics libraries utilizing alkylation-based sample preparation. In line with other researchers,^34,35^ we suggest using protein libraries covering all naturally occurring amino acids in their free form and assessing the effect of the most common modifications typical of each target amino acid identified in the sample.

Notwithstanding these constraints, the post-Cys specificity uncovered in this study opens up new opportunities for omitting Cys blocking by alkylation in *An*PEP/neprosin-driven proteolysis. Since *An*PEP is specific at low pH, the risk of accidental disulfide bond formation is avoided, so samples can be fully reduced at neutral-basic pH and then rapidly acidified before analysis, preventing disulfide bond reformation and enabling digestion after Cys residues. On the contrary, for applications where higher *An*PEP/neprosin specificity is crucial, Cys alkylation becomes another key parameter to consider, together with the enzyme/protein ratio, pH, and digestion time.

The role of these proteases in the treatment of digestive disorders should be reassessed because *An*PEP and neprosin can target Cys. The enzymatic mechanism of PPCEs should also be revisited based on these results. PPCEs can cleave not only after Pro and Ala but also after Cys, and in the light of this extended selectivity, the less prominent cleavages after small, nonbranched (Gly and Ser), and basic (Lys and Arg) amino acids should be considered as well.

After a comprehensive and targeted search for literature on post-Cys cleavage specificity PPCEs, we only found a single conference paper suggesting this selectivity for human prolyl endopeptidase and dipeptidyl peptidases.^36^ Because their post-Cys cleavage has remained unnoticed by the proteomics field, we must reassess the impact of this activity on regulatory mechanisms in the human body^36^ and reconsider both PPCEs^13,17^ applications and mechanisms of action,^13,16^ thereby opening new opportunities in protein analysis.

## Conclusions

PPCEs like *A. niger* prolyl endopeptidase and neprosin target not only Pro and Ala but also reduced Cys. This cleavage preference can be blocked by various modifications, such as Cys alkylation, disulfide bonding, and oxidation, and explains why post-Cys specificity has remained unnoticed despite extensive research of these enzymes. Moreover, post-Cys specificity seems to be a common feature of proteases targeting Pro and Ala. Under strictly controlled conditions and with more complex samples, *An*PEP produces a specific digestion pattern, albeit not transferable to individual proteins, less complex samples, or highly abundant proteins in otherwise complex mixtures. In such samples, *An*PEP will most likely show lower specificity.

## Data Availability

All primary MS data and secondary data (*.mgf, *.csv, databases) have been deposited to ZENODO under DOI: https://dx.doi.org/10.5281/zenodo.13938580 and https://dx.doi.org/10.5281/zenodo.13985598.

## Supporting Information Available

SDS-PAGE analysis of different *An*PEP sources; cleavage preferences for online digestion of protein mixture and serum samples on an *An*PEP column; cleavage preferences at P1 and P1' site for in-solution digestion of protein mixture comparing *An*PEP and ProAlanase; and cleavage preferences at P1 site for in-solution digestion of protein mixture by *An*PEP at different pH provided as separate files (PDF)
List of peptides used to generate graphs in Figure 1 (XLS)
List of proteins identified in LC–MS/MS profiling of *An*PEP (XLS)
Quantitative estimation of proteins identified in LC–MS/MS profiling of *An*PEP (XLS)
