# Supplementary material for: Postproline Cleaving Enzymes also Show Specificity to Reduced Cysteine
Source: Anal Chem. 2024 Nov 19;96(48):19084–92. doi: 10.1021/acs.analchem.4c04277 (PMC11618732; doi:10.1021/acs.analchem.4c04277)
Supplement: Supplementary file 1 — ac4c04277_si_001.pdf [file ac4c04277_si_001.pdf]

Supporting Information

Postproline Cleaving Enzymes also Show Specificity to Reduced Cysteine

Zuzana Kalaninová[†,‡], Jasmína Mária Portašiková[†,‡], Barbora Jirečková[†,‡], Marek Polák[†,‡], Jana Nováková[§], Daniel Kavan[‡], Petr Novák[†,‡], and Petr Man[*,‡]

[†]Department of Biochemistry, Faculty of Science, Charles University, Hlavova 6, Prague 2, 12843, CZ;
[‡]Institute of Microbiology of the Czech Academy of Sciences, BioCeV, Videnska 1083, Prague 4, 14220, CZ;
[§]AffiPro s.r.o., Nad Safinou II 366, Vestec, 252 00, CZ

The content in supporting information

1. Figure S1 – SDS-PAGE analysis of two *An*PEP sources
2. Figure S2 – Cleavage preferences based on online digestion of a protein mixture and serum samples on an *An*PEP column
3. Figure S3 – Cleavage preferences at the P1' site based on in-solution digestion of a protein mixture comparing *An*PEP and ProAlanase at different digestion times and enzyme/protein ratios
4. Figure S4 – Cleavage preferences at P1 and P1' site based on in-solution digestion of a protein mixture comparing *An*PEP and ProAlanase at 2-hour-digestion time and different enzyme/protein ratios
5. Figure S5 – Cleavage preferences at the P1 site extracted based on digestion of a protein mixture at pH 1.5 and pH 4.5 for 2 h and 18 h
6. Table S1 – Lists of peptides used to generate Figure 1 and Figure S2 graphs (provided as a separate *.xls file - ***ac4c04277_si_002.xls***)
7. Table S2 – List of proteins identified in the LC-MS/MS profiling of *An*PEP Clarity Ferm (provided as a separate *.xls file - ***ac4c04277_si_003.xls***)
8. Table S3 – Quantitative estimation of proteins identified in the LC-MS/MS profiling of *An*PEP Clarity Ferm (provided as a separate *.xls file - ***ac4c04277_si_004.xls***)

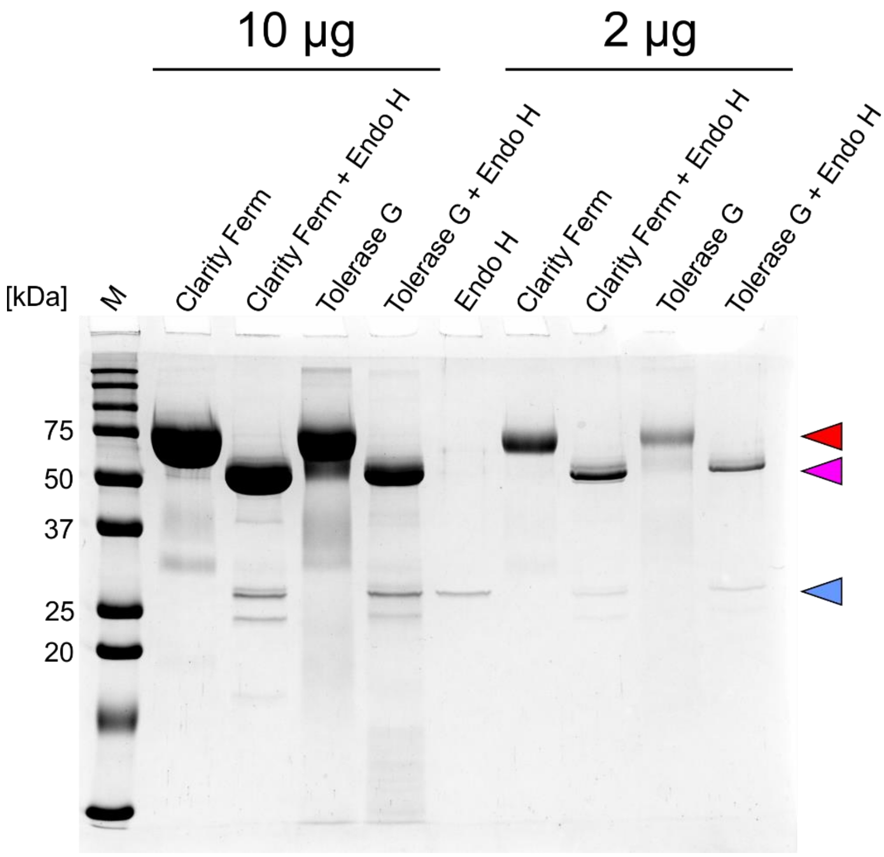

Figure S1. SDS-PAGE analysis of two different *An*PEP sources - Clarity Ferm and Tolerase G – before (red triangle) and after (pink triangle) deglycosylation with EndoH (blue triangle). Both sources show high purity/homogeneity, with only minor contaminations and are highly N-glycosylated. Two protein amounts, 10 µg and 2 µg, were used, as indicated above the gel.

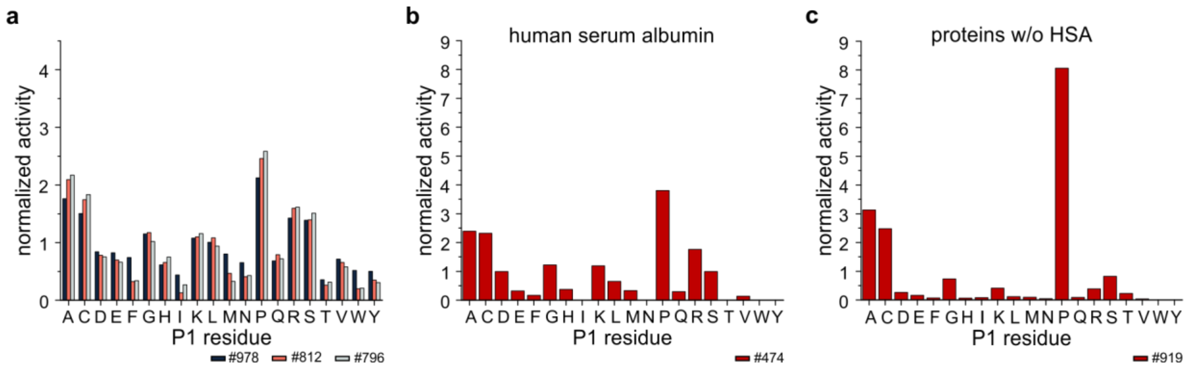

Figure S2. Cleavage preferences based on online digestion of: (**a**) a protein mixture on an *An*PEP column, considering all identified peptides and (**b**) pre-reduced human serum using data extracted for human serum albumin only or (**c**) all identified proteins excluding serum albumin.

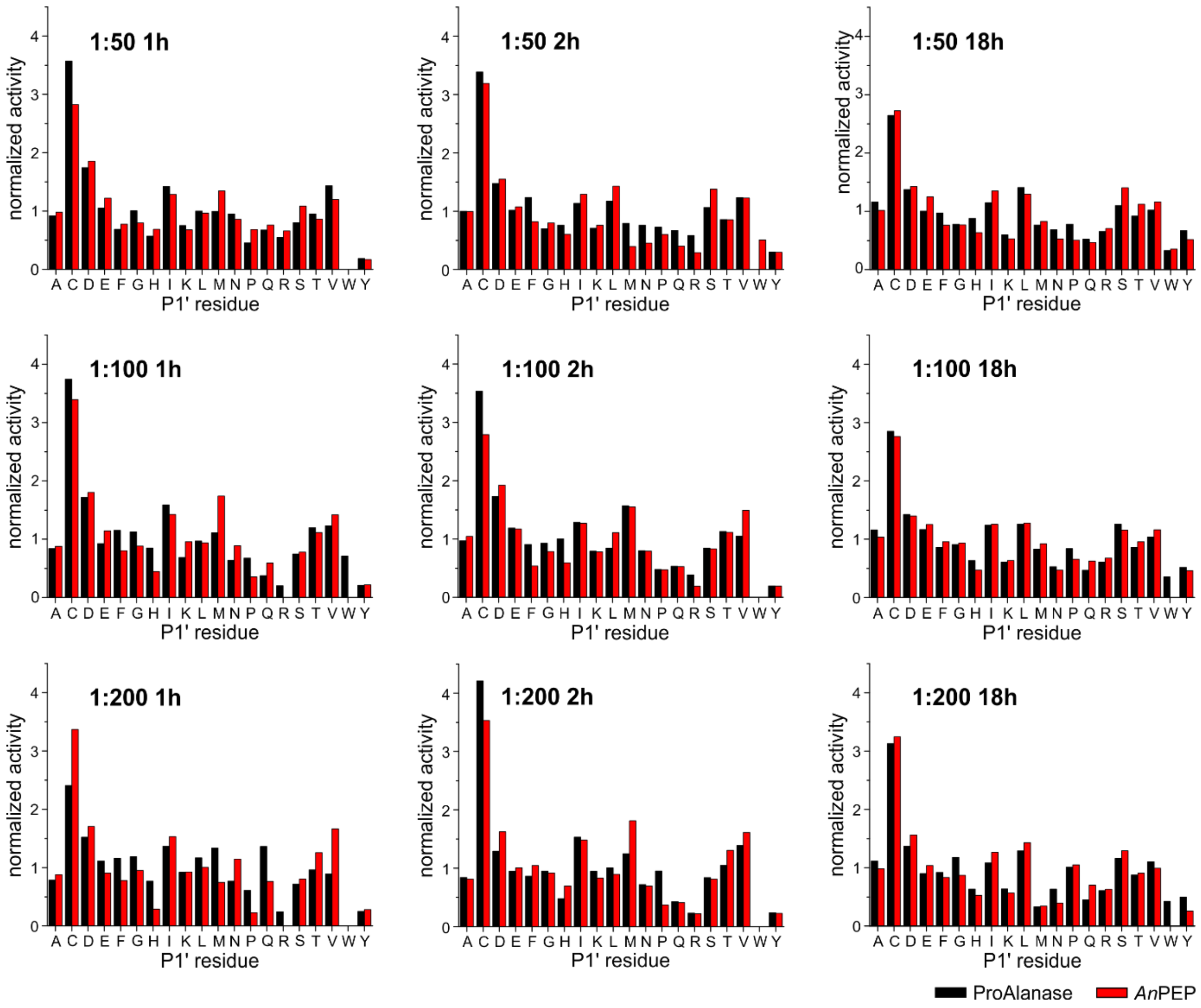

Figure S3. Cleavage preferences at the P1‘ site based on in-solution digestion of a protein mixture comparing *An*PEP and ProAlanase at different digestion times (1, 2 and 18 h) and enzyme/protein ratios (1:50, 1:100 and 1:200). All graphs are normalized to the same value at the y-axis.

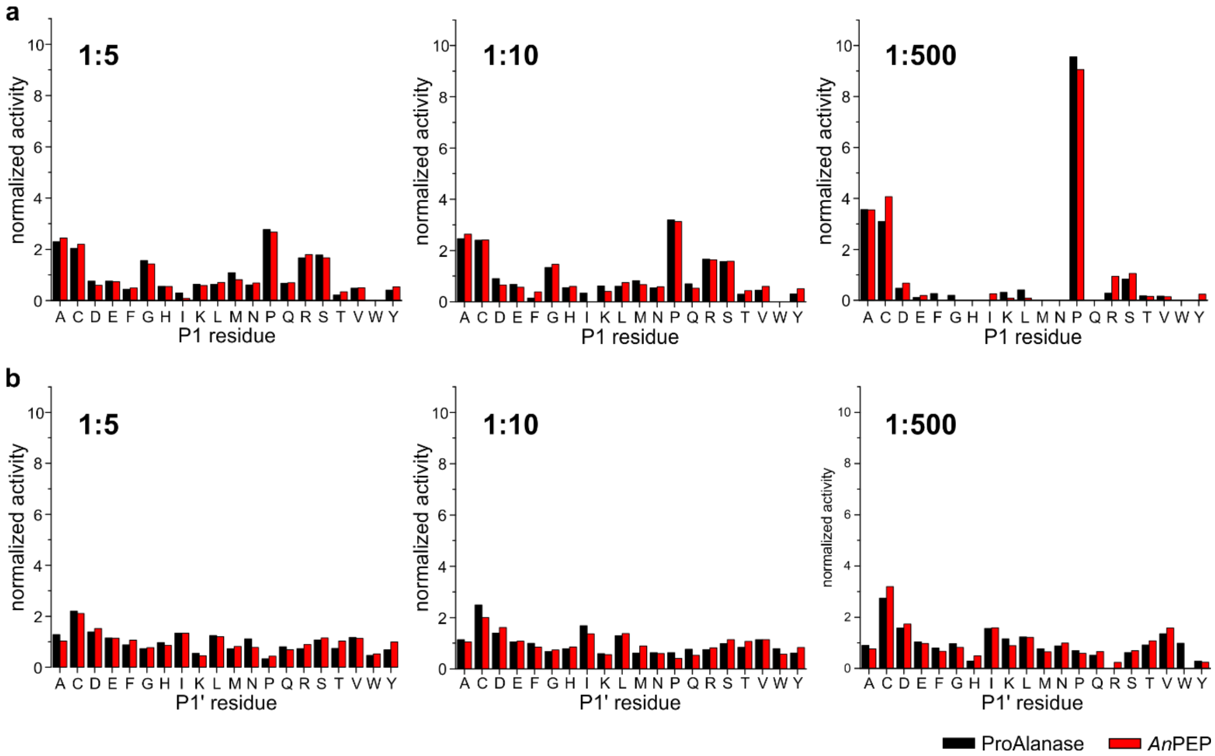

Figure S4. Cleavage preferences at P1 (**a**) and P1’ (**b**) site based on in-solution digestion of a protein mixture comparing *An*PEP and ProAlanase at 2-hour-digestion time and 1:5, 1:10 and 1:500 enzyme/protein ratios. All graphs are normalized to the same value at the y-axis.

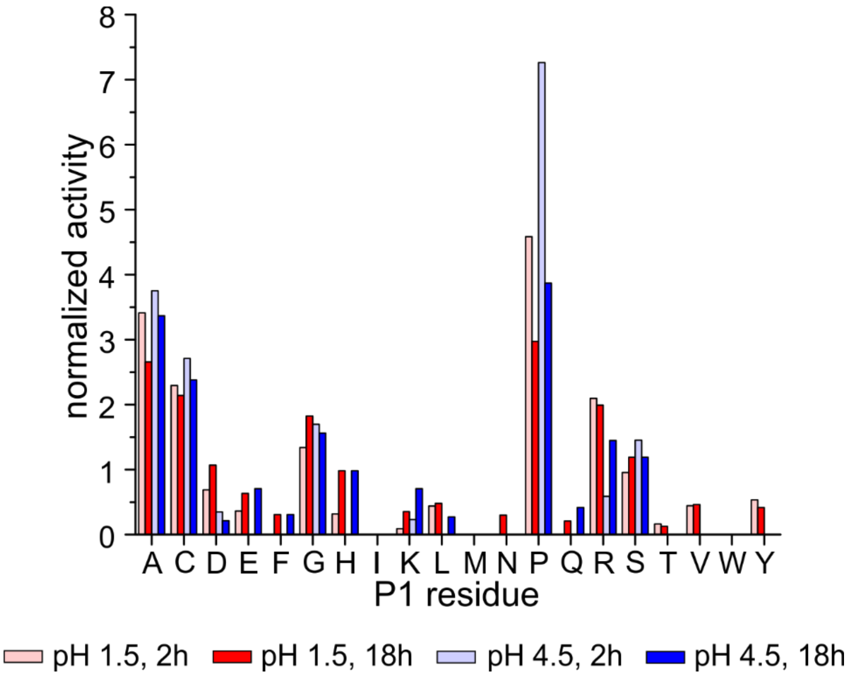

Figure S5. Cleavage preferences extracted from digestion at pH 1.5 (red tones) and at pH 4.5 (blue tones) for 2 h and 18 h. Digestion after Cys is observed at both pH values. In contrast, cleavage after Asp is suppressed at higher pH, indicating the clear contribution of acid hydrolysis to post-Asp cleavage at low pH.
